# Whole genome sequence analysis of *Cupriavidus campinensis* S14E4C, a heavy metal resistant bacterium

**DOI:** 10.1007/s11033-020-05490-8

**Published:** 2020-05-13

**Authors:** Gorkhmaz Abbaszade, Attila Szabó, Balázs Vajna, Rózsa Farkas, Csaba Szabó, Erika Tóth

**Affiliations:** 1 Department of Microbiology, Institute of Biology, Eötvös Loránd University, Budapest, Hungary; 2 Lithosphere Fluid Research Lab, Institute of Geography and Earth Sciences, Eötvös Loránd University, Budapest, Hungary

**Keywords:** *Cupriavidus campinensis*, Whole genome sequence, Heavy metal resistance

## Abstract

*Cupriavidus* sp*.* are model organisms for heavy metal(loid) resistance and aromatic compound’s degradation studies and these characteristics make them a perfect candidate for biotechnological purposes. Bacterial strain S14E4C (identified as *Cupriavidus campinensis*) was isolated from a playground by enrichment method in a 0.25 mM containing medium. The analysis revealed that this bacterium is able to tolerate high concentrations of heavy metal(loid)s: Cd up to 19.5 mM, Pb to 9 mM, Hg to 5.5 mM and As to 2 mM in heavy metal(loid) salt containing nutrient medium. The whole genome data and analysis of the type strain of *C. campinensis* CCUG:44526^T^ have not been available so far, thus here we present the genome sequencing results of strain S14E4C of the same species. Analysis was carried out to identify possible mechanisms for the heavy metal resistance and to map the genetic data of *C. campinensis*. The annotation pipelines revealed that the total genome of strain S14E4C is 6,375,175 bp length with a GC content of 66.3% and contains 2 plasmids with 295,460 bp (GC content 59.9%) and 50,483 bp (GC content 63%). In total 4460 coding sequences were assigned to known functions and 1508 to hypothetical proteins. Analysis proved that strain S14E4C is having gene clusters such as *czc, mer, cus, chr, ars* to encode various heavy metal resistance mechanisms that play an important role to survive in extreme environments.

**Electronic supplementary material:**

The online version of this article (10.1007/s11033-020-05490-8) contains supplementary material, which is available to authorized users.

## Introduction

Extensive use of metals and chemicals in the industrial processes have resulted in accumulation of large quantities of effluents containing toxic heavy metals in the environment, and these effluents pose environmental disposal problems due to their non-degradable and persistent characters [1]. In biological systems, heavy metals have been reported to interact with cell components such as DNA and different proteins, causing DNA damage and block functional groups of important molecules or transport channels. Hence, microorganisms have evolved unique characteristics to tolerate/resist heavy metals by various mechanisms, such as detoxification, bioprecipitation, bioaccumulation, etc., which proved to be an ideal tool in bioremediation of heavy metal contaminated environments. Therefore, understanding the impact of heavy metal(loid)s on microorganisms and mechanisms of metal resistance is crucially important and imperative in order to remove and recover heavy metals from polluted environments.

*Cupriavidus* is a genus of the family *Burkholderiaceae* that is well known for its heavy-metal resistance and diverse metabolic capabilities in different niches, especially from heavy metal and organic-chemical contaminated soils [2]. The β-proteobacterium *Cupriavidus* (formerly *Wautersia*, *Ralstonia*) *campinensis* was isolated first time from Campine, the geographical region of northeast Belgium [3]. This bacterium was found to be highly resistant to heavy metals [3] and following analysis confirmed the ability of degradation 2,4-dichlorophenoxyacetic acid by other strain [4] as well, as the extrachromosomal genetic determinants were transferable to related bacteria. In the present study the whole genome of *Cupriavidus campinensis* strain S14E4C was sequenced and analysed in detail, as well as heavy metal resistance genes and genomic potentials were characterised.

## Materials and methods

### Isolation of the bacterial strain

*C. campinensis* S14E4C was isolated from the heavy metal contaminated playground (GPS coordinates: 48°05′42.0″N 19°47′32.8″E) of Salgótarján (Hungary), a former industrial city. Sample was obtained by scraping away the upper (grassy) surface of the soil with a sterile knife and the upper 10–15 cm thick soil layer was placed into sterile plastic tubes, then transported to the laboratory at 4 °C temperature condition. The sample was enriched with 0.25 mM of Cd^2+^ salt containing broth (DSM medium 1, without agar) by constantly shaking (270 rpm) for two weeks at room temperature. Isolation of strains was performed in a random manner from the growing colonies by standard dilution plate technique. Bacterial strain S14E4C was maintained at 28–30 °C on nutrient agar medium (DSM medium 1) supplemented with 0.25 mM cadmium (Cd).

### DNA isolation and identification

Identification of strain S14E4C was done based on 16S rRNA gene sequencing: the genomic DNA of strain S14E4C was extracted using a DNA extraction kit (DNeasy Power Lyzer Microbial Kit, Qiagen, Germany). To confirm the identities of the isolates the 16S rRNA gene was amplified (PCR) from the extracted genomic DNA using the universal primers 27f (5′-AGAGTTTGATCCTGGCTCAG-3′) and 1492r (5′-GGCTACCTTGTT ACGACTT-3′) [5] (LGC Genomics, Berlin, Germany). The 16S rRNA gene sequence of strain S14E4C was compared with references in the EzTaxon database [6] and the NCBI Nucleotide database using BLAST [7] to identify closely related bacteria.

### Genome sequencing and assembly

The whole genome shotgun and paired-end sequencing of the strain S14E4C was performed by the Genomics Facility RTSF, Michigan State University (USA), on an Illumina MiSeq platform using the MiSeq standard v2 chemistry. Low quality reads, with excess “N” and low quality score, duplication reads, and adaptor contamination were filtered out from the sequence set. Subsequently, high quality reads were assembled using the SPAdes v3.10.0 assembler in careful mode [8] and the existence of plasmids in the genome was identified by plasmid SPAdes (v3.5.0) tool [9]. The assembly quality was checked by QUAST v2.3 [10] and coverage was calculated by coverage calculator master v0.0.1 (https://github.com/GenomicaMicrob/coverage_calculator).

### Genome annotation and analysis

Genome annotation, prediction of genome features and functions were analysed by various tools, such as RAST (Rapid Annotation using Subsystem Technology) [11], PATRIC 3.5.38 [12] and DDBJ Fast Annotation and Submission Tool (DFAST) [13] web interfaced pipelines. The annotation results of tools were combined in order to cover throughout the genome. Additionally, after submission the NCBI Prokaryotic Genome Annotation Pipeline (PGAP) (https://www.ncbi.nlm.nih.gov/genome/annotation_prok/) annotated the genome. Functional genes that were investigated as having possible roles in metabolic pathways were checked by KEGG database [14] on PATRIC 3.5.38. Phylogenetic classification of proteins encoded in the S14E4C genome were based on clusters of orthologues group (COG) functions [15].

### Determination of minimum inhibitory concentration (MIC) and antibiotic resistance

MIC values of the 4 heavy metal(loid)s (Cd, Hg, Pb and As) for *C. campinensis* S14E4C were determined using nutrient medium (DSM medium 1) supplemented with the following heavy metal(loid) salts (CdSO_4_, HgCl_2_, Pb(NO_3_)_2_ or As_2_O_3_, respectively). Analysis has started with 0.25 mM of relevant salt concentrations and after one week the adapted cultures transferred to elevated concentrations (1.5–9 mM for Pb, 0.65–19.5 mM for Cd, 1.46–5.5 mM for Hg, 1–2 mM for As) media. Additionally, heavy metal tolerance of the strain S14E4C was checked by low phosphate Tris-salt mineral medium with heavy metal salt additives (CdSO_4_, HgCl_2_, Pb(NO_3_)_2_ or As_2_O_3_,) in various concentrations (1.5–2.4 mM for Pb, 0.65–2 mM for Cd, 1.5–1.85 mM for Hg, 0.5–1 mM for As).

Antibiotic resistance of strain S14E4C was tested on nutrient agar medium using disk diffusion method of EUCAST regulation (www.eucast.org) Version 8.0 (January 2020).

### Degradation of aromatic compounds

The strain was tested for its ability to degrade phenanthrene and naphthalene as aromatic compounds. Cultivation was performed in a 48 ml sterile Bushnell Haas Broth (BHB) containing 2 ml bacterial suspension (50 μl trace element solution, and 1 mg/l phenanthrene and 5 mg/l naphthalene) in sterile 100 ml sealed serum bottle at 24 °C on a magnetic stirrer. Three replicates were tested and the variations were measured after 3 and 7 days by using SPME GC-FID [16–18].

### Phylogenetic analysis

To determine the phylogenetic relationships among *Cupriavidus* species, the public and completed 16S rRNA gene sequences of the corresponding *Cupriavidus* type strains were gathered from the Arb-Silva database [19] and aligned by SINA 1.2.11 aligner in SILVA ACT (Alignment Classification and Tree Service) service before creating Maximum Likelihood (ML) tree. A rooted phylogenetic tree based on 16S rRNA gene sequence similarity of the genera *Cupriavidus* was created using CIPRES Science Gateway’s MrBayes tool [20] and the closely related bacterium *Polynucleobacter cosmopolitanus* CIP 109840^T^ (AJ550672) was used as an outgroup. Phylogenetic tree was visualized by FigTree v1.4.4 [21]. In addition, PATRIC [12] presents the reference and representative genomes and uses them as part of the Comprehensive Genome Analysis in phylogenomic analysis. The closest reference and representative genomes have been identified by Mash/MinHash [22]. In order to determine the phylogenetic position of this genome, PATRIC chose global protein families (PGFams) [23] from these genomes. Then these protein sequences were aligned with MUSCLE [24] and the nucleotides were plotted to the protein alignment of each these sequences. The amino acid and nucleotide alignments were linked into a data matrix, and RaxML tool [25] was used to analyse this matrix using quick bootstrapping [26] and produce support values in the tree.

## Results and discussion

Since the first isolation of *C. campinensis* [3]*,* though some of the metabolic pathways and characteristics were revealed, most of them are still unknown due to the lack of whole genome information of the species. Whereas, genome sequence of *C. campinensis* S14E4C revealed the strain has additional capabilities, such as heavy metal resistance, degradation of aromatic compounds, antibiotic resistance, etc. that enhance its potential for use in biotechnological applications. To predict open reading frames and similarity of the species several annotation and alignment programs were used (PATRIC, RAST, DFAST) and compared to be able to detect genes with high accuracy.

It is indicated that *C. campinensis* S14E4C adapts and resists to the effect of environmental stresses by functional genes and moreover, genes related to plasmid partitioning and the plasmid initiating (protein *RepA*) are solid evidence for plasmid existence that perform heavy metal and antibiotic resistance. Through combination of sequencing and comparative genomics our study has provided, for the first time, a comprehensive genomic description of the species *Cupriavidus campinensis*.

### Genomic data of *Cupriavidus campinensis* S14E4C

The 16S rRNA gene sequence of strain S14E4C (NCBI GenBank accession MK660715) was obtained and BLAST search results based on EzTaxon and GenBank databases both indicated that strain S14E4C belongs to the genus *Cupriavidus* and it is 100% identical to *Cupriavidus campinensis* WS2 [3]. The Whole Genome Shotgun project of strain S14E4C has been deposited at DDBJ/ENA/GenBank under the accession VCIZ00000000. The version described in this paper is version VCIZ01000000. The strain S14E4C has been accessioned into the National Collection of Agricultural and Industrial Microorganisms under the accession number NCAIM B.02650.

### Genome structure and general features of *C. campinensis* strain S14E4C

The genome of S14E4C is 6,322,653 bp with a GC content 66.3% after assembly to 52 contigs (contigs shorter than 500 bp were removed) with 78.3 × coverage value. Total of 5968 putative coding sequences (CDSs) were validated by homology and 4460 CDSs that were assigned to one or more function classes (Table 1), whereas, 1508 CDSs are identified as a hypothetical based on function annotation. The draft genome contained 49 tRNA and 7 rRNA genes (including 5S, 16S and 23S rRNA).

**Table 1:** General features of *Cupriavidus campinensis* S14E4C genome

Features	Genome	Plasmid 1	Plasmid 2
Size (bp)	6,322,653	295,460	50.483
Contigs	52	4	7
Contig L50	7	1	1
Contig N50	290,832	212,313	43,173
GC content	66.3	59.9	63
tRNA	49	0	6
rRNA	7	0	3
Total number of CDSs	5968	351	43
CDSs with assigned functions	4460	158	27
Hypothetical proteins	1508	193	16
CDSs with EC number assignments	1265	31	9
CDSs with GO assignments	1091	27	9
CDSs with KEGG pathway assignments	972	17	9

Previous studies depicted that known members of the *Cupriavidus* genus contain 2 large replicons (generally a chromosome and a chromid) and several plasmids [2, 27–32]. In the current study the replicons were identified by the PlasmidSPAdes v.3.5 software that the algorithm using contigs’ read coverage information, estimates median coverage, builds assembly graph and generates plasmidic contigs [9]. The analysis resulted in 2 plasmids with the length of 295,460 bp and 50,483 bp, and with the average GC content of 59.9% and 63%, respectively. To validate, the obtained results, the draft genome sequence (52 contigs) and putative chromids/plasmids of S14E4C strain were aligned by Mauve v.2.4 software [33] with whole genome, chromosome and plasmid sequences of the known *Cupriavidus* species (mainly *C. metallidurans*) (Supplementary Fig. 1). The alignments clarified the identical genes in Plasmid1 of the S14E4C, exist in both plasmids of the *C. metallidurans* CH34, whereas only few positions are similar among plasmid 2 and CH34 plasmids (Supplementary Fig. 1, C and D). After the annotation of the plasmid sequences, it is assumed that in the strain S14E4C, the main replicon carried most of the essential housekeeping genes, including those for translation, ribosome production, DNA replication, DNA repair, protein processing, cell component and resistance. Plasmid 1 contains genes mainly encoding mechanisms of heavy metal resistance (cadmium, mercury, copper, zinc, etc.) and membrane cation transport, additionally genes encoding proteins involved in carbohydrate metabolism and c-type cytochrome biogenesis. Whereas, plasmid 2 carries genes for antibiotic resistance (e.g. *tetMOPQST)* and operon for ribosomal protein synthesis (SSU rRNA, LSU rRNA, 5S rRNA). The existence of the rRNA operon (*rrn*) on plasmids and chromids was earlier reported on *Bacillus* and *Paracoccus* species [34] as well, due to its functional importance.

### Genome annotation

#### Metabolic pathways

**Fig. 1:**
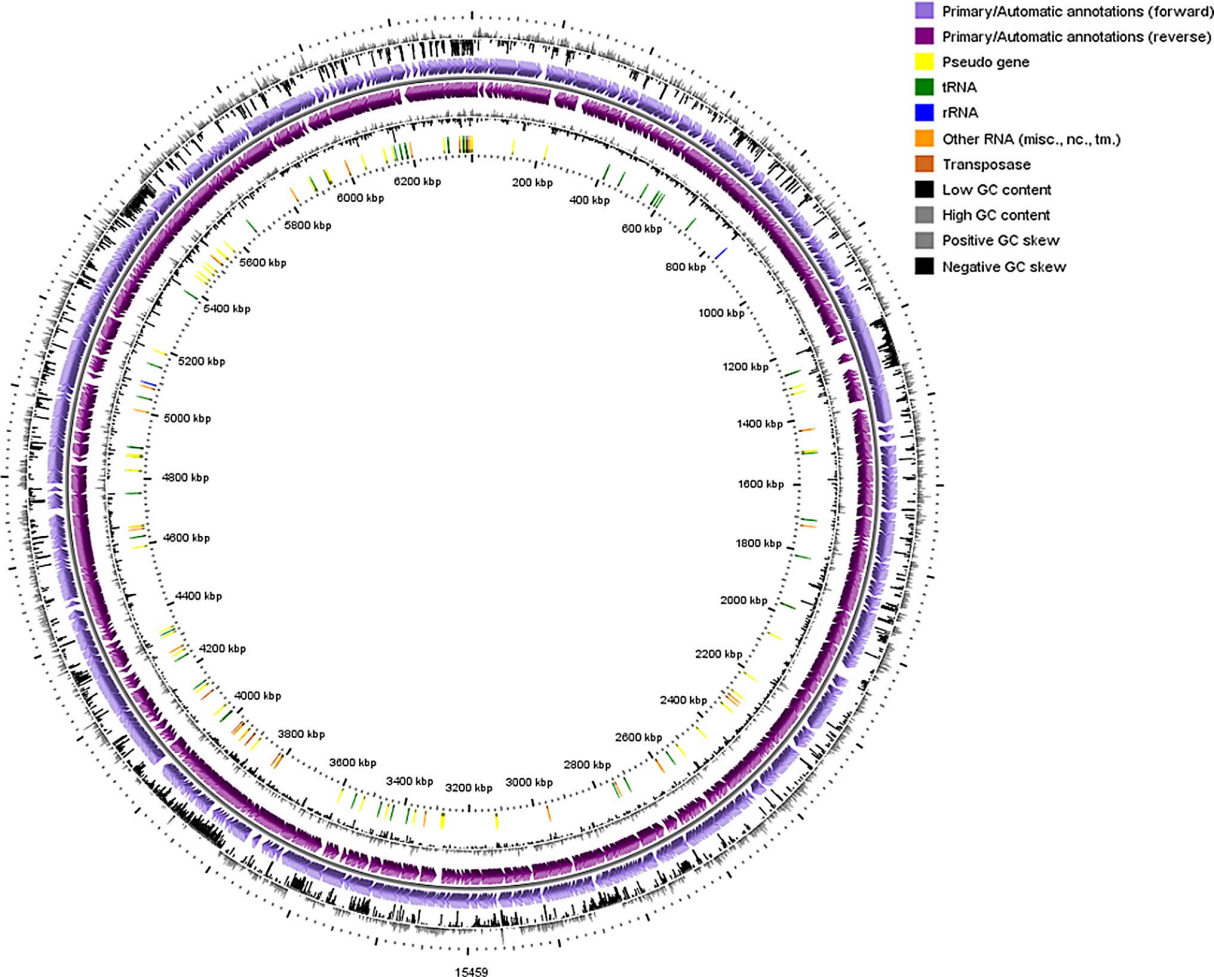
A circular graphical display of the genome (contains chromosome and plasmid contigs) and applicable genes. This includes CDS on the forward strand, CDS on the reverse strand, RNA genes, Transposase, pseudogene, GC content and GC skew. The figure was prepared by CGView circular genome visualization tool [62]. (Color figure online)

The genome of *C. campinensis* S14E4C consists mostly of known genes encoding metabolic modules and various pathways support its growth. The main and common metabolic genes were shown in Fig. 1. Strain S14E4C just as many bacteria has genetic capacity for nitrogen, sulfur, phosphorus and different carbohydrates metabolism. Among them genes of cyanate hydrolysis (*cynRXST* operon), nitrate and nitrite ammonification (*nrf, nar, nit, nat* reductase or transport), nitrate reductase (*narRKGHJIA, nirVK, norDQBCFE, nosXLYFDZR*) and gene clusters responsible for nitrogen metabolism are also present in its genome.

The biochemistry of the bacterial sulfur metabolism pathways is quite complex and encoded by *soxABXYZDFCRSWH* gene cluster. Basically, sulfur oxidation pathways require only five *sox* genes whose products form three key periplasmic protein complexes: *soxYZ*, a sulfur carrier protein, *soxXA*, a c-type cytochrome complex, and *soxB*, a sulfate thiol hydrolase [28]. In case of organic sulfur assimilation, alkanesulfonate assimilation and utilization occurs by *ssuA*—alkanesulfonates-binding, *ssuB*—alkanesulfonate ABC transporter ATP binding, s*suF*—organosulfonate utilization, *ssuC*—alkanesulfonates transport system permease, *ssuD*—alkanesulfonate monooxygenase, etc. proteins.

**Fig. 2:**
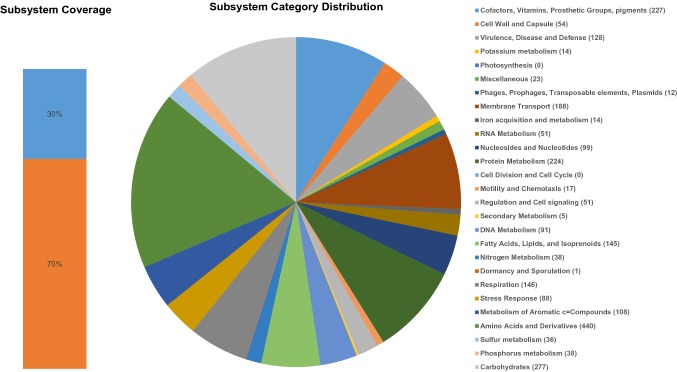
Subsystem coverage and category distribution of whole genome. The pie chart demonstrates the counts for each subsystem feature and the subsystem coverage. Genes for each Subsystem Category were shown in brackets. (Color figure online)

S14E4C implements phosphate metabolism with *ptsS* (putuative periplasmic phosphate binding protein), *ptsA* (phosphate transport system permease protein), *ptsB* (phosphate transport ATP binding protein), *oprO* and *oprP* (pyrophosphate and phosphate specific outer membrane porins) genes (Fig. 2).

Spectrum of carbohydrate metabolism is broad, but here only few of them are mentioned. Such as, several operons are responsible for maltose and maltodextrin utilization (*malEFGKMPRAZ*) and mannose metabolism (*manYZBCEFGKL, mtpEFGKL*). However, based on the annotation results, compared to previously identified *Cupriavidus* species the metabolism of ketogluconates (some can serve as the sole source of carbon and energy for various bacteria) is quite different in the *Cupriavidus campinensis* S14E4C.

In *C. campinensis* strain S14E4C the process proceeds via conversion to 6-phosphogluconate, which is further metabolized through the Entner-Doudoroff (EDP) and/or Pentose Phosphate pathways (PPP). The pathway is encoded by *pglDH* gene, whereas, there is no gene for 6-phosphogluconate dehydrogenase (6-PGDH) in any earlier sequenced *Cupriavidus* species [28] (e.g. *C. metallidurans, C gilardii, C. necator*, etc.).

### Genes/gene clusters of heavy metal(loid) resistance (HMR)

Referring to the genome annotation analysis *Cupriavidus campinensis* strain S14E4C possesses extensive number of heavy metal(loid) resistant genes and gene clusters (Table 2). Comparing to the metal resistant bacterium *Cupriavidus metallidurans* CH34, strain S14E4C carries many typical metal resistance clusters, such as *czcABC*, *copCBA*, etc.[28]. However, for various metal tolerant strains metal resistance mechanisms can differ slightly. For instance, alternate cation-specific mechanisms of Zn^2+^, Cd^2+^ and Co^2+^ resistance and transport encoded by the same genes (*czcRDABC*) [35].

**Table 2:** Genes/gene clusters of heavy metal(loid) resistance (HMR) in *Cupriavidus campinensis* strain S14E4C

Genes	Functions	Metal(loid)	Locus tag
*czcA*^*# 12*^	Cobalt-zinc-cadmium resistance protein	Cd^2+^, Co^2+^, Zn^2+^	FGG12_RS19525
*czcB*^*# 12*^	Cobalt-zinc-cadmium efflux RND transporter, membrane fusion protein	Cd^2+^, Co^2+^, Zn^2+^	FGG12_RS19530
*czcC*^*# 12*^	Heavy metal RND efflux outer membrane protein	Cd^2+^, Co^2+^, Zn^2+^	FGG12_RS19535
*czcR*^*# 12*^	Cobalt-zinc-cadmium resistance protein	Cd^2+^, Co^2+^, Zn^2+^	FGG12_RS19515
*czcE*^*# 12*^	CzcE family metal-binding protein	Cd^2+^, Co^2+^, Zn^2+^	FGG12_RS19505
*czcI*^*# 12*^	Cobalt-zinc-cadmium regulatory protein	Cd^2+^, Co^2+^, Zn^2+^	FGG12_RS19540
*cadA*^*# 12*^	Cadmium-translocating P-type ATPase	Cd^2+^, Co^2+^, Zn^2+^	FGG12_RS19635 FGG12_RS19680
*cadR*^*# 12*^	Cobalt-zinc-cadmium transcriptional regulatory protein	Cd^2+^, Co^2+^, Zn^2+^	FGG12_RS19620
*merA*^*# 12*^	Mercuric ion reductase	Hg^2+^	FGG12_RS19735
*merC*^*# 12*^	Mercuric transport protein	Hg^2+^	FGG12_RS19740
*merP*^*# 12*^	Periplasmic mercury (+2) binding protein	Hg^2+^	FGG12_RS19745
*merT*^*# 12*^	Mercuric transport protein	Hg^2+^	FGG12_RS19750
*merR*^*# 12*^	Mercuric resistance operon regulatory protein	Hg^2+^	FGG12_RS19755
arsB*^1^	Arsenic transporter (efflux pump)	As^3+^, As^5+^	FGG12_RS00815
arsC*^1^	Arsenate reductase	As^5+^	FGG12_RS25020 FGG12_RS00810
arsR*^1,4,6^	Transcriptional regulator	As	FGG12_RS00835 FGG12_RS01270 FGG12_RS11920 FGG12_RS09700
arsH*^1^	Organoarsenical detoxification	As^3+^	FGG12_RS00805
*chrA*^*32*^	Chromate transport protein (RND efflux)	Cr^6+^	FGG12_RS28790
*copC*^#^*^1, 12^	Copper resistance protein	Cu^2+^	FGG12_RS19835 FGG12_RS03095
*copD*^#^* ^*1, 12, 16*^	Copper resistance protein	Cu^2+^	FGG12_RS19840 FGG12_RS23205 FGG12_RS03090
*copG*^*# 12*^	CopG family transcriptional regulator		FGG12_RS19865
*copQ *^*2,3,5*^	Copper resistance protein	Cu^2+^	FGG12_RS04775 FGG12_RS07055 FGG12_RS08045 FGG12_RS10890
*copK*^*# 12*^	Periplasmic Cu(I)/Cu(II)-binding protein CopK		FGG12_RS19800
*cusA*^*2,3,5,8*^	Cation efflux system protein	Cu^2+^, Ag^+^	FGG12_RS04660 FGG12_RS15640 FGG12_RS08075 FGG12_RS11275
*cutA *^*13*^	Periplasmic divalent cation tolerance protein	Cu^2+^	FGG12_RS20295
*cueR*^*12,23*^	Cu(I)-responsive transcriptional regulator	Cu^+^	FGG12_RS19675 FGG12_RS26765
*hupE*^*17*^	UreJ family metal transporter	Ni^2+^	FGG12_RS23365
*corA *^*4,13,16,22*^	Magnesium and cobalt transport protein	Mg^2+^, Co^2+^	FGG12_RS23065 FGG12_RS20305 FGG12_RS26445 FGG12_RS09045
*mgtC *^*2,4,12*^	Mg^2+^ transport ATPase protein	Mg^2+^	FGG12_RS19555 FGG12_RS04485 FGG12_RS09090

Some genes exist in multiple copies and locations were shown on each cell

^#^Genes located on plasmid

*Genes located on chromosome; Numbers depict relevant contigs; Locus tags are from NCBI annotation

**Fig. 3:**
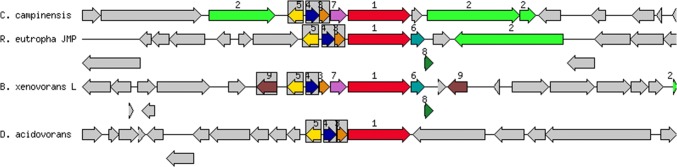
Mercury resistance gene cluster: The chromosomal region of the focus gene (top) is compared with three similar organisms. The graphic depicts the focus gene, which is red and numbered 1. Sets of genes with similar sequence are grouped with the same number and colour (1- mercuric ion reductase *merA*; 2- TnpA transposase (left), Transposase Tn3 (right); 3- periplasmic mercury (2 +) binding protein *merP*; 4- mercuric transport protein *merT*; 5- transcriptional regulator *merR* family; 6- mercuric resistance operon coregulator *merD;* 7- mercuric transport protein *merC;* 8- mercuric transport protein *merE;* 9- DNA-invertase). Genes whose relative position is conserved in at least three other species are functionally coupled and share grey background boxes

**Fig. 4:**
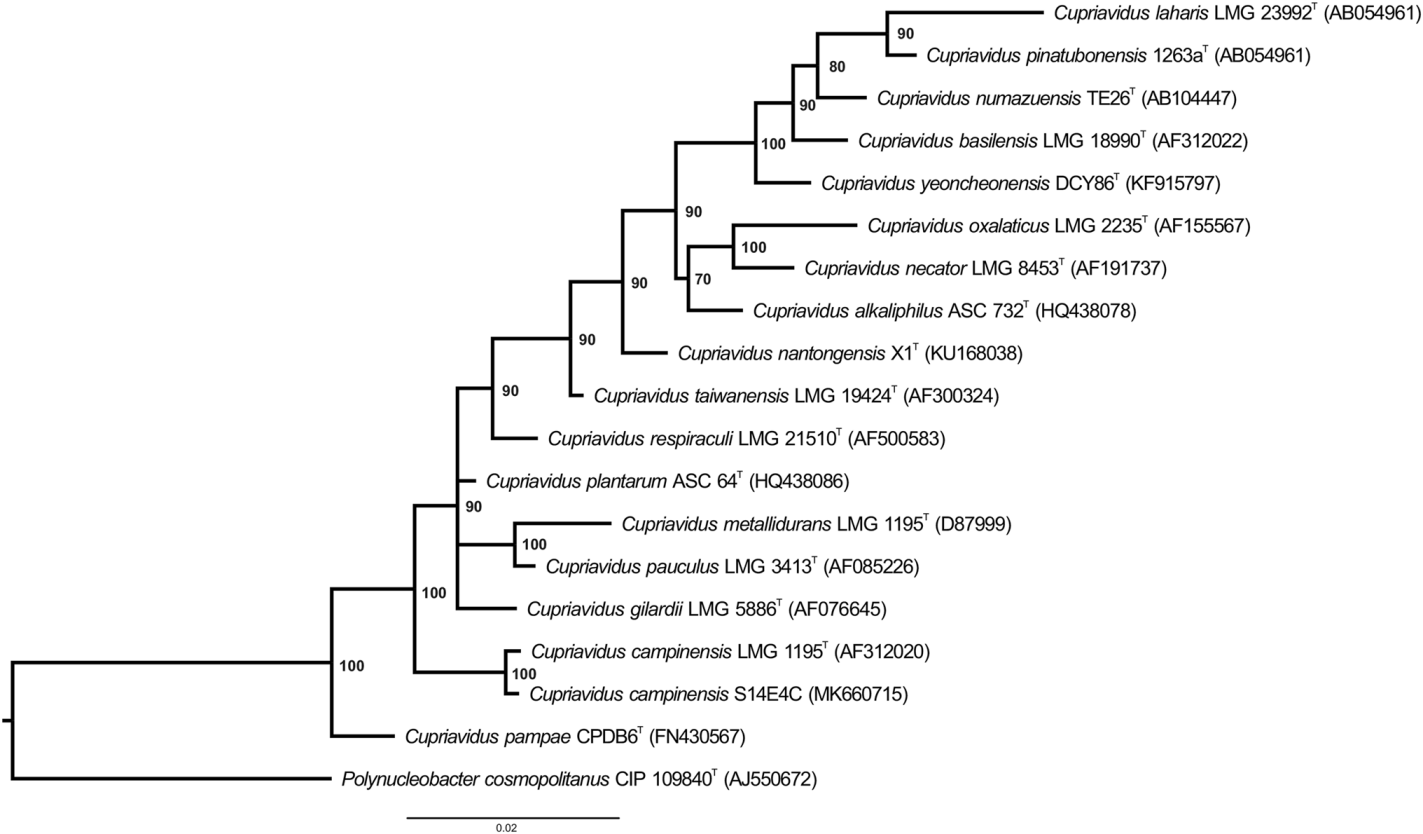
Phylogenetic relationship of *Cupriavidus campinensis* S14E4C strain and the species of Cupriavidus based on 16S rRNA gene sequence. Cluster analysis was based upon the neighbour-joining method with *Polynucleobacter cosmopolitanus* CIP 109840^T^ (AJ550672) as the outgroup root. The MrBayes method were used to generate the tree and its support values (only values above 50% are presented). Bar, 0.02 substitutions per nucleotide position. Tree was visualised by FigTree v1.4.4

The genome also carries system genes and clusters involved in the transport and resistance of Cd^2+^, Pb^2+^, Ni^2+^, Co^2+^, such as *nccAB*, *nikABCDEKLMNOQR*, *cbtACDFGJKLX*, *ctpD*, plasmid-mediated *trcD*, etc. Unlike the CH34, pbr operon is absent in S14E4C, but previous researches indicate that heavy metal-(Cd, Co, Pb, Zn)-translocating P-type ATPase genes can perform Pb^2+^ resistance as well [36]. However, the mechanisms of Hg and As resistance is different than Cd, Pb, Zn resistance. Mercuric ions are toxic to bacteria because they bind to sulfhydryl groups and hinder macromolecule synthesis and enzymatic functions. The Hg^2+^ resistance system at strain S14E4C consisted of *merR* gene, activates transcription of *mer* operon (*merRTPCADE*) in elevated concentrations of mercury, and genes encode the resistance to mercury (Hg) is a well-known property of both Gram-positive and Gram-negative bacteria that generally locate in plasmids [37, 38]. The operon is located on contig 12 and delimited by transposon sequences (Figs. 3, 4). The *merC*, *merT*, *merE* and *merP* genes function as membrane or periplasmic transport of organic and inorganic Hg^2+^ (Table 2). The *merA* and *merB* genes (*merB*) responsible for demethylation of organic mercury compounds by cleavage C-Hg bonds, encoding mercuric reductase and the enzyme organomercurial lyase directly followed by genes encoding transport and transcriptional regulators. The other gene encoding organomercury resistance, *merD*, a secondary regulatory protein, also binds the same region as *merR*, involved in transcriptional regulation [39, 40].

In addition to the cluster elements, several *ars* gene homologs (*arsB, arsC, arsR, arsH*) were identified in the genome of *Cupriavidus campinensis* S14E4C for arsenate (AsO_4_^3−^) resistance and the strategy followed by bacteria depend on the arsenate reductase (ArsC) protein. In the existing operon (*arsRBC)* the *arsC*, arsenate reductase is able to transform the arsenate to arsenite and the rest of the process is encoded by *arsB*, an integral membrane protein, to prohibit arsenic accumulation by expelling out of the cytoplasm [41, 42]. Meanwhile, the metalloregulatory protein ArsR (encoded by *arsR*) enables the transcription of the operon by attaching the promoter region [43, 44]. The strain can oxidize methyl arsenate compounds (by *arsH* gene) that contributes to the global biotransformation of arsenic [45]. Among the bacterial species, the *arsH* is widely distributed in Proteobacteria (not present in Gram-positive) and similarly to other *ars* genes locates mostly in chromosome [44, 46].

Encoded proteins for the efflux system of cations including chromate (CrO_4_^2−^) are distributed in the genome of S14E4C as a cluster *chrABCEIF*, while, available gene clusters, *copACDSRZ*, *cusSARBCF, scsABCD, cutACEF and ycnLKI* are encoding proteins that responsible for the uptake, efflux, translocation and periplasmic detoxification of Cu^+^ and Cu^+2^ ions. The protein sequences of *cusCFBA* are similar to the general gene and protein families which pump Cu^2+^ cations out of cells that had been previously studied. Additionally, except the Cu^2+^ cations, the *cusCBA* genes are encoding Ag^+^ efflux system as well (Table 2). Another gene responsible for Ag^+^ resistance is putative silver efflux pump (SEP*)*. In the environment Cu^2+^ is more abundant and less toxic than Cu^+^ and the presence of protein *copA*, P-type Cu^+^ efflux ATPase, controlling the intracellular Cu^+^ level, whereas, the extracellular periplasmic space is defended by *cusCFBA* multicomponent efflux transport system [47]. The chemiosmotic carrier *cusCFBA* RND efflux has been examined in details [47] and the *cusF* protein displayed as a key periplasmic copper binding protein [48].

It can be concluded that metal(loid) detoxification in *C. campinensis* S14E4C primarily occurs by efflux system(s): RND (Resistance, Nodulation, cell Division) family of permeases, the CDF (Cation Diffusion Facilitator) family of heavy metal transporters and the P-type ATPase family of ion pumps may operate on a variety of cations [49], including Cd^2+^, Zn^2+^, Pb^2+^, Ni^2+^, CrO_4_^2−^, Ag^+^, Co^2+^, Cu^2+^, Cu^+^, Hg^2+^, and likely on other heavy metals (Table 2).

### MIC (Minimum Inhibitory Concentration) results of *Cupriavidus campinensis* S14E4C

In order to verify metal resistance capability of the strain S14E4C MIC assessment was performed and *C. campinensis* S14E4C expressed high tolerance/resistance to all four tested heavy metals. The nutrient and Tris-salt mineral medium indicated significant difference in terms of growth inhibition (Table 3). Resistance to heavy metal(loid)s in nutrient media was extremely high most probably due to the adaptation period. Meanwhile, though resistance to HM of the strain S14E4C in low phosphate Tris-salt mineral medium was lower, these values are not considerably high (2 mM Cd^2+^, 2.4 mM Pb^2+^, 1.84 mM Hg^2+^, 1 mM As^3+^) compared to other *Cupriavidus* species, though the mercury resistance is noticeable (1.84 mM).

**Table 3:** Comparison of *Cupriavidus* species Minimum Inhibitory Concentration values

MIC (mM)^−1^					
Metal ionic form	Compound^b^	*C. gilardii* CR3	*C. metallidurans* CH34	*C. campinensis* S14E4C	*C. campinensis* S14E4C
Cd^2+^	CdCl_2_*5H_2_O	4	4	–	–
	CdSO_4_*8H_2_O	–	–	19.5	2
Hg^2+^	HgCl_2_	> 0.04	0.0027	5.5	1.84
Pb^2+^	Pb(NO_3_)_2_	4	1	9	2.4
As^3+^	As_2_O_3_	–	4	2	1
Used media		Tris-buffered mineral salt medium	Tris-salt mineral medium	Nutrient medium	Tris-salt mineral medium

^a^MIC values of *C. gilardii* CR3 and *C. metallidurans* CH34 are from reference [28, 30, 50], *C. campinensis* is from this study

^b^C. gilardii CR3 and C. metallidurans CH34 cadmium resistance was checked by CdCl_2_*5H_2_O, whereas in *C. campinensis* S14E4C by CdSO_4_*8H_2_O salt

### Antibiotic resistance

The Genome Annotation Service in PATRIC uses k-mer-based ABR genes detection method [12] that assigns antibiotic resistance genes and their functions, though, the existence of ABR-related genes in a genome does not directly mean antibiotic resistance phenotypically. A summary of the ABR genes annotated in this genome and related ABR mechanism [51] is given in Table 4. On the basis of genome sequencing results *C. campinensis* S14E4C is resistant to D-cysteine amino acids and Beta-lactam group antibiotics with around 21 resistance genes. D-cysteine is decomposed into pyruvate, H_2_S, and NH_3_ by D-cysteine desulfohydrase. Decomposition of D-cysteine by the desulfohydrase (*dcyD* gene) produces H_2_S, which the bacterium can use as a sulfur source. In S14E4C strain the desulfhydrase appears in a cluster with two proteins (CAP, CPP*)*, probably involved in transport processes, and a gene encoding a periplasmic cystine-binding protein (CBP). Additionally, *blc, bli, blR, blaI, blaR* resistance genes exist in the genome of the S14E4C for resistance of β-lactam group antibiotics. These antibiotics include penicillin, cephalosporin, cephamycin and carbapenem, supply multi-resistance by breaking the antibiotics’ structure. *C. campinensis* S14E4C carries β-lactam resistance genes that responsible for penicillin binding, repression, hydrolase and regulation [52] to have become effective at their function as antibiotic resistance enzymes. The inner membrane chemiosmotic pump protein *acrB* and a substitute for the bridging protein *acrA* are the homologs of Pseudomonas *mexA* and *mexB* [53], where *C. campinensis* S14E4C contains all of these 4 genes. Other ABR genes were shown on Table 4.

**Table 4:** Antibiotic resistance genes (ABR) found in *C. campinensis* strain S14E4C by PATRIC 3.5.38 annotation pipeline

ABR mechanism	Genes
Antibiotic activation enzyme	*katG*
Antibiotic target in susceptible species	*alr, ddl, dxr, ef-g, ef-tu, folA, dfr, folP, gyrA, gyrB, inhA, fabI, iso-tRNA, kasA, murA, rho, rpoB, rpoC, s10p, s12p*
Antibiotic target protection protein	*bcrC*
Efflux pump conferring antibiotic resistance	*emrAB-OMF, emrAB-TolC, mdtABC-OMF, mdtABC-tolC, mexAB-oprM*
Gene conferring resistance via absence	*gidB*
Protein altering cell wall charge conferring antibiotic resistance	*gdpD, pgsA*
Regulator modulating expression of antibiotic resistance gene	*h-ns, oxyR*

The test with 9 antibiotic agents revealed that the strain S14E4C can resist to the number of antibiotics (Table 5). In 2006, it was reported by Baker-Austin and co-workers that heavy metal pollution increases the metal resistance and reduce antibiotic sensitivity due to co-regulation of genes [54, 55]. Hence, we assume that the isolation of S14E4C from the heavy metal polluted industrial city promoted the heavy metal and antibiotic resistance as well.

**Table 5:** Resistance of S14E4C to antibiotic compounds

Antibiotic (μg)	Disk diffusion test (inhibition zone diameter—mm)	Gradient MIC test (mg/l)
Penicillin (1U)	6	> 32
Cefuroxim (30)	35	–
Ampicillin (10)	8	16
Ceftriaxon (30)	38	0.5
Ceftazidim (10)	22	2
Imipenem (10)	35	0.5
Meropenem (10)	6	> 32
Aztreonam (30)	12	64
Cefoxitin (30)	25	8

Active ingredient content of antibiotic discs indicated in brackets

Disk diffusion: 6 mm – no inhibition zone (exact marking ≤ 6 mm)

### Metabolism of aromatic compounds

Annotation results identified also the presence of genes encoding enzymes involving in the utilization of various aromatic compounds in *C. campinensis* S14E4C as a source of carbon and energy. The pathway genes that are found in this strain include, benzoate degradation (*benBACDKEF* operon) and transport (*bt1254* transporters) [56], salicylate ester degradation (*salARED*, *areABCR*), additionally, gentisate catabolism and degradation, N-heterocyclic aromatic compounds degradation (OQD, IQOb), etc.

The degradation capacity of our strain was measured connected to phenanthrene and naphthalene and after one week of incubation it was not detectable. It is assumed that the isolation environment (not contaminated by aromatic compounds) of the strain plays direct role in these results, however, the genes for the possible degradation of aromatic compounds could be activated after a long time exposure [57, 58].

### Phylogenetic analysis

**Fig. 5:**
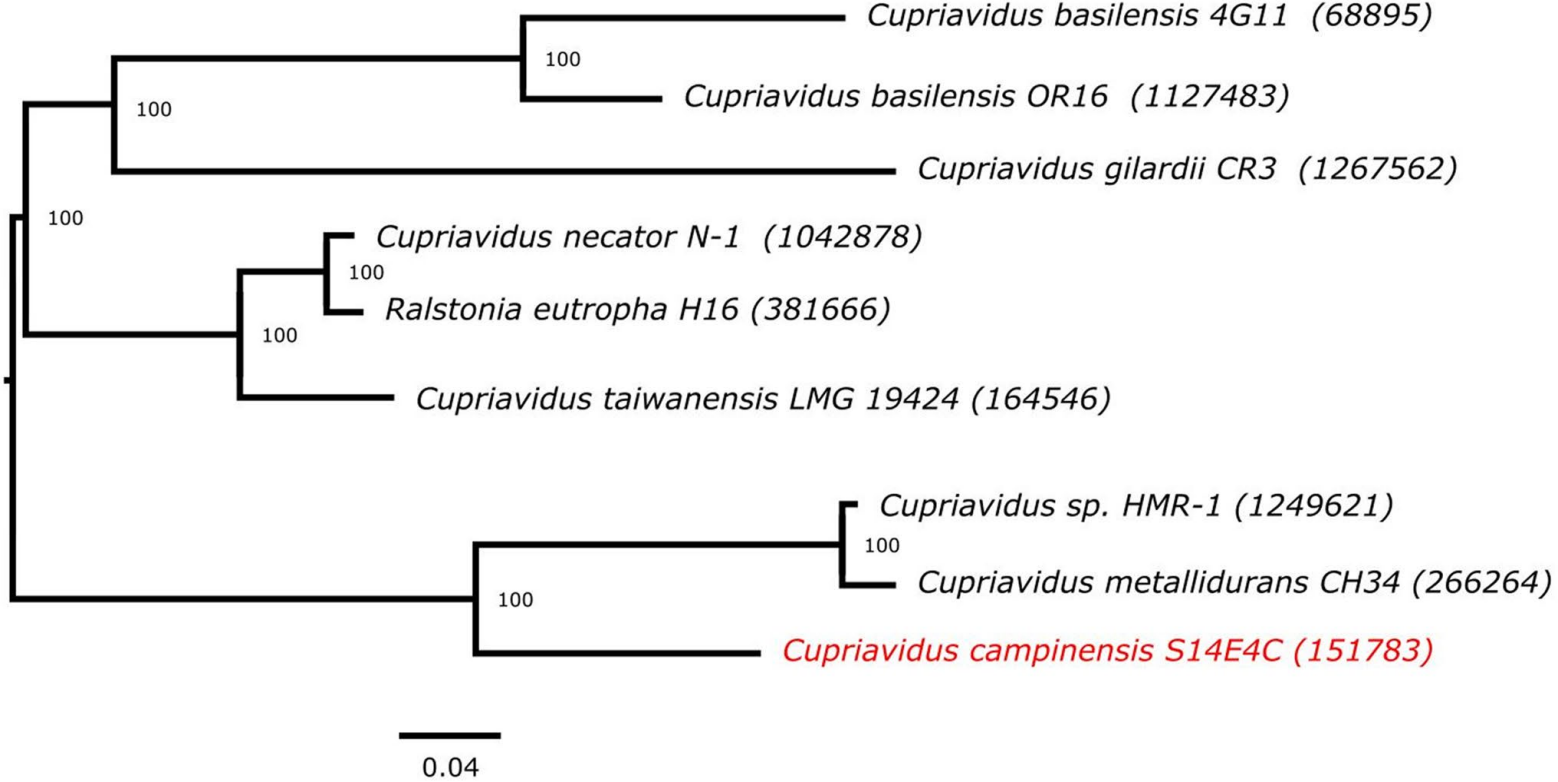
Phylogenomic tree of the *Cupriavidus campinensis* S14E4C based on concatenation of 16S rRNA gene with core genes (e.g. gyrB, rpoD, recA, etc.). Tree was built on PATRIC online pipeline

**Fig. 6:**
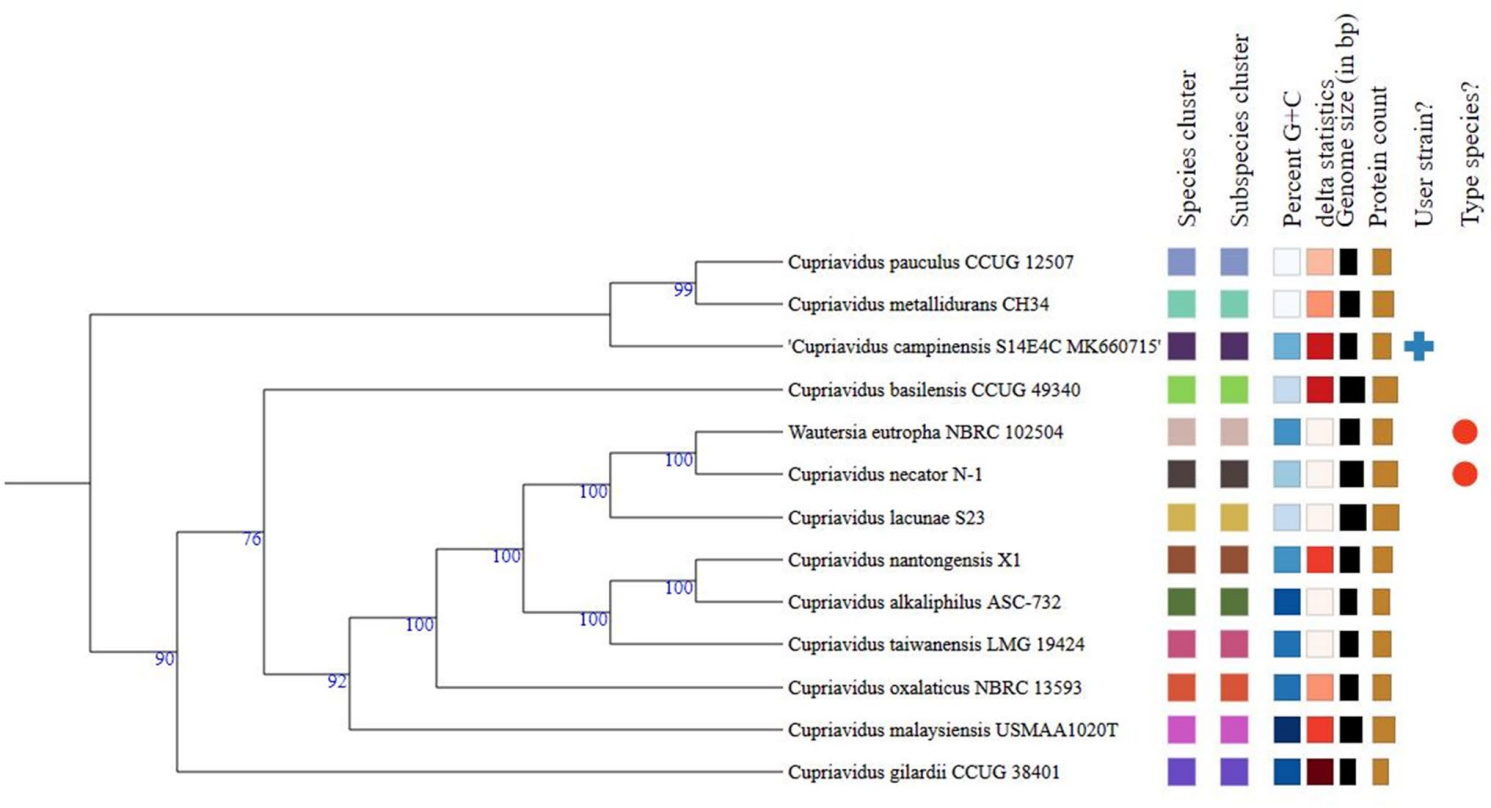
Phylogenomic tree predicted on TYGS database. Genomic G + C content (63.53–68.47%), δ values (0.08–0.2), overall genome sequence length (5,783,696–9,185,558 bp), number of proteins (5142–7932). Values increase based on the colour range (from white to black) [59]

Phylogenetic analysis based on 16S rRNA gene sequence suggested that *C. campinensis* S14E4C strain is a member of *Cupriavidus* genus and its closest relative was *C. campinensis* LMG 1195^T^ (AF312020) (Fig. 5). Besides, strain S14E4C phylogenetically close to *C. gilardii and C. pampae* with 98% similarity value. Additionally, some other core genes (e.g. *gyrB, rpoD, recA*, etc.) were concatenated with the 16S rRNA gene and analysed on PATRIC [12]. In fact, the precise phylogenetic position of *C. campinensis* S14E4C was placed and depicted in Fig. 5 and these results supported by the tree generated on TYGS database (based on genome signatures) that depicted similar results (Fig. 6) [59]. The genome-wide Average Nucleotide Identity (gANI) value between strain S14E4C and *C. metallidurans* CH34 was identified [60] as 81.98% to confirm the genomic relatedness. This method suggests firm resolution amidst closely linked genomes (80–100% ANI) [61].

## Electronic supplementary material

Below is the link to the electronic supplementary material.

Supplementary Figure 1. Syntheny plot analysis of the S14E4C sequence vs known Cupriavidus species and their replicons. (A) Syntheny plot of C. campinensis S14E4C vs C. gilardii CR3, C. necator N-1, C. metallidurans CH34 (PNG 433 kb)

Supplementary Figure 1. Syntheny plot analysis of the S14E4C sequence vs known Cupriavidus species and their replicons. (B) Chromosome from C. metallidurans CH34 vs C. campinensis S14E4C whole genome (PNG 433 kb)

Supplementary Figure 1. Syntheny plot analysis of the S14E4C sequence vs known Cupriavidus species and their replicons. (C) Megaplasmid from C. Metallidurans CH34 vs C. campinensis S14E4C whole genome (PNG 433 kb)

Supplementary Figure 1. Syntheny plot analysis of the S14E4C sequence vs known Cupriavidus species and their replicons. (D) Plasmid pMOL28 and pMOL30 from C. metallidurans vs plasmid 1 and plasmid 2 from C. campinensis S14E4C (PNG 433 kb)
