# Supplementary figures and images for: Whole genome sequence analysis of Cupriavidus campinensis S14E4C, a heavy metal resistant bacterium
Source: Mol Biol Rep. 2020 May 13;47(5):3973–85. doi: 10.1007/s11033-020-05490-8 (PMC7239810; doi:10.1007/s11033-020-05490-8)

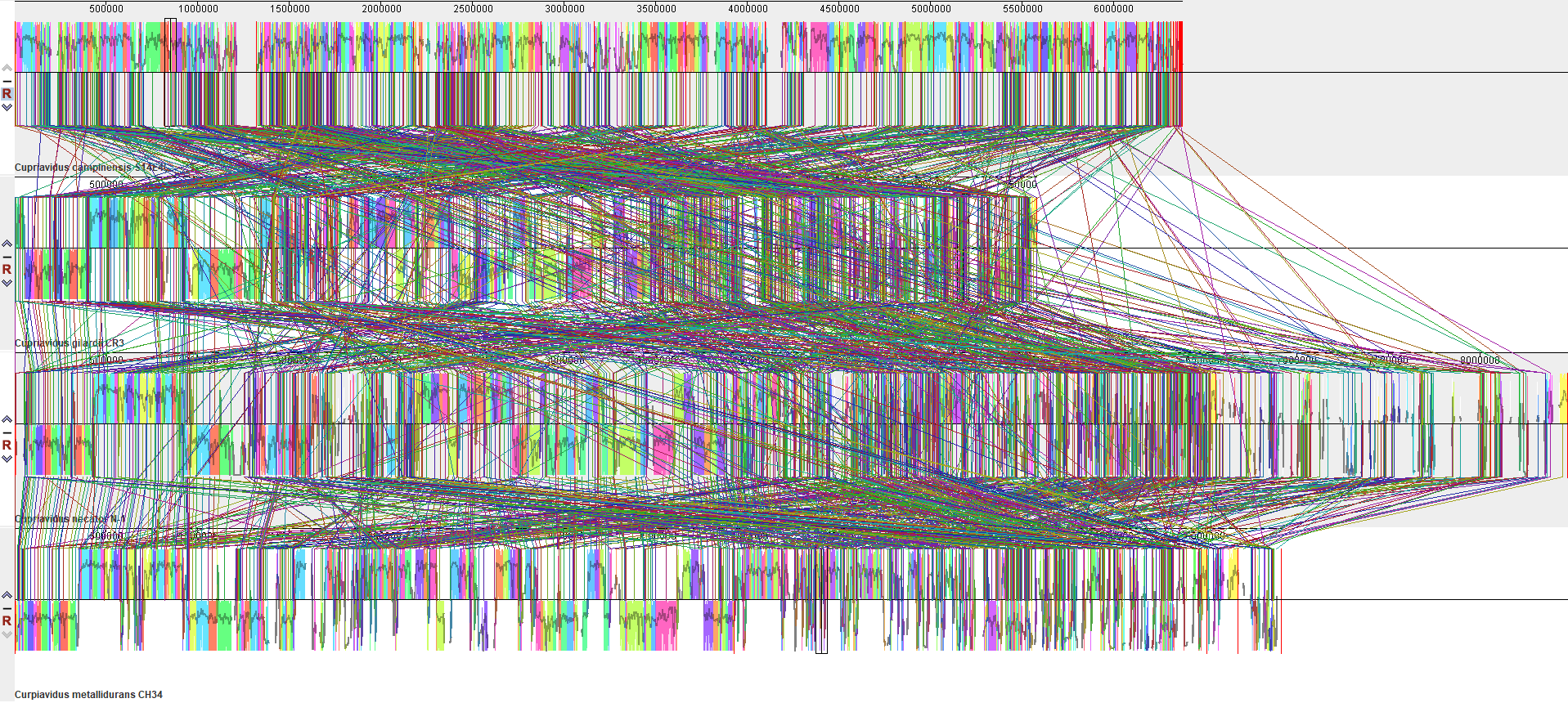

Supplement: Supplementary file 1 — Supplementary Figure 1. Syntheny plot analysis of the S14E4C sequence vs known Cupriavidus species and their replicons. (A) Syntheny plot of C. campinensis S14E4C vs C. gilardii CR3, C. necator N-1, C. metallidurans CH34 (PNG 433 kb) [file 11033_2020_5490_MOESM1_ESM.png]

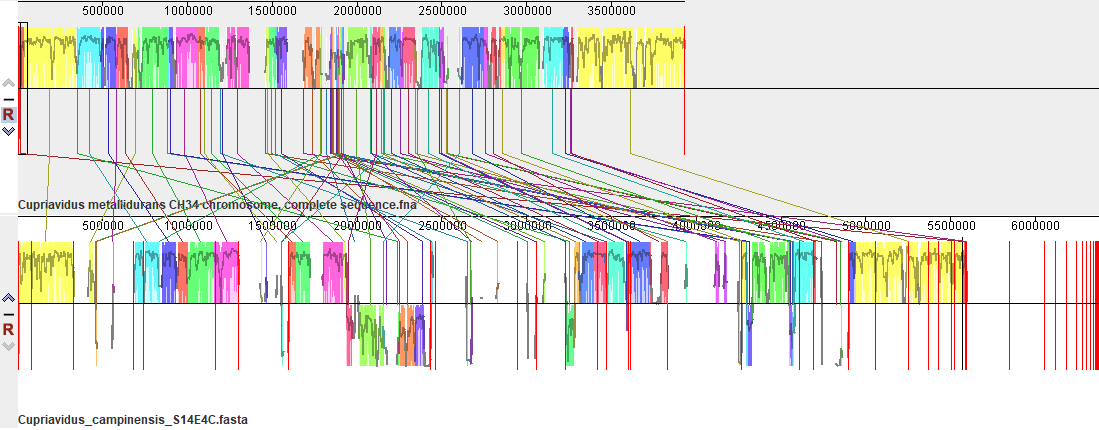

Supplement: Supplementary file 2 — Supplementary Figure 1. Syntheny plot analysis of the S14E4C sequence vs known Cupriavidus species and their replicons. (B) Chromosome from C. metallidurans CH34 vs C. campinensis S14E4C whole genome (PNG 433 kb) [file 11033_2020_5490_MOESM2_ESM.png]

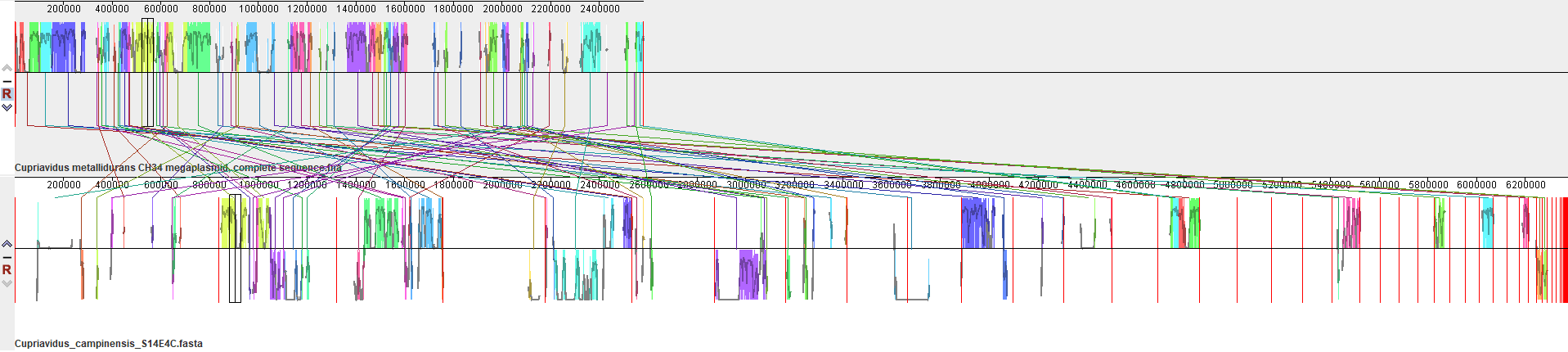

Supplement: Supplementary file 3 — Supplementary Figure 1. Syntheny plot analysis of the S14E4C sequence vs known Cupriavidus species and their replicons. (C) Megaplasmid from C. Metallidurans CH34 vs C. campinensis S14E4C whole genome (PNG 433 kb) [file 11033_2020_5490_MOESM3_ESM.png]

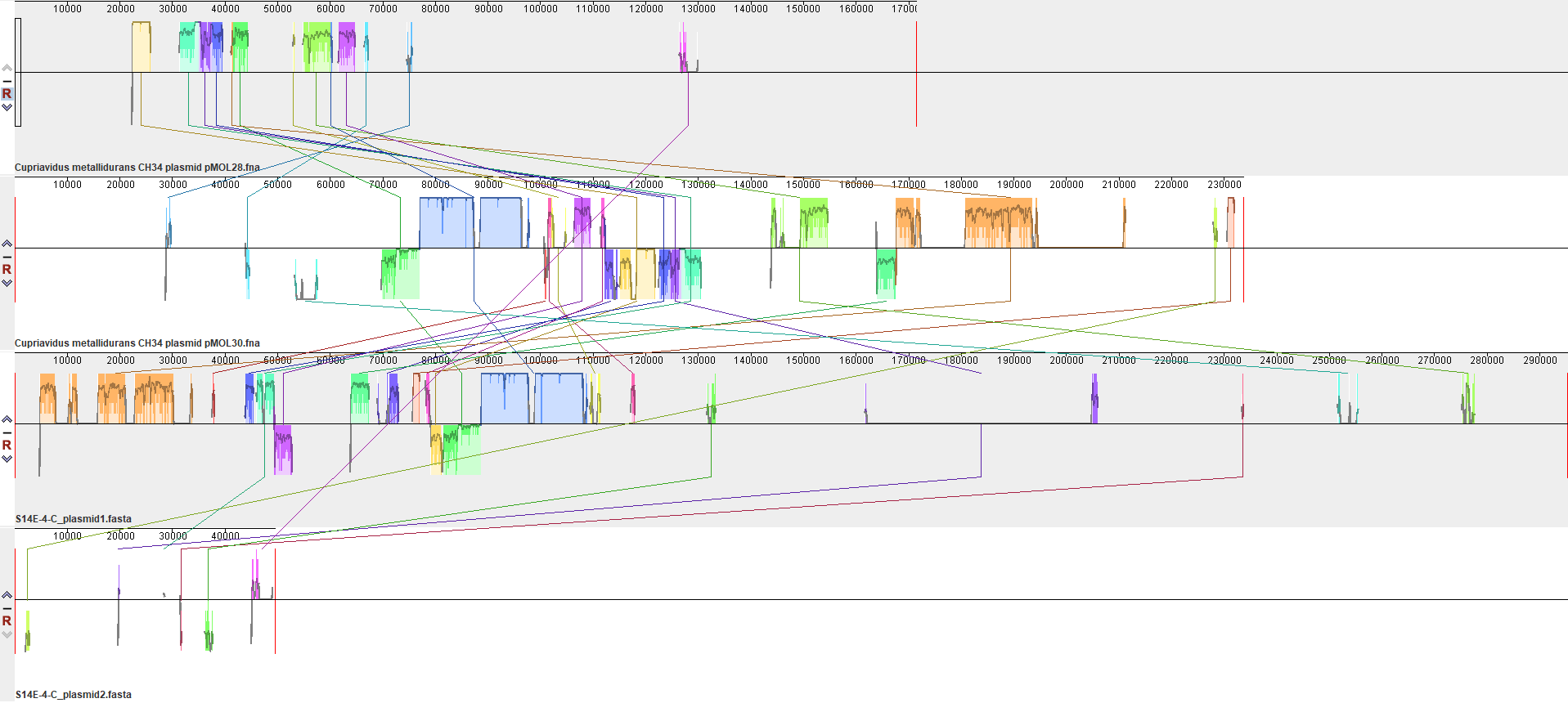

Supplement: Supplementary file 4 — Supplementary Figure 1. Syntheny plot analysis of the S14E4C sequence vs known Cupriavidus species and their replicons. (D) Plasmid pMOL28 and pMOL30 from C. metallidurans vs plasmid 1 and plasmid 2 from C. campinensis S14E4C (PNG 433 kb) [file 11033_2020_5490_MOESM4_ESM.png]
